# Category Selectivity of Human Visual Cortex in Perception of Rubin Face–Vase Illusion

**DOI:** 10.3389/fpsyg.2017.01543

**Published:** 2017-09-12

**Authors:** Xiaogang Wang, Na Sang, Lei Hao, Yong Zhang, Taiyong Bi, Jiang Qiu

**Affiliations:** ^1^Key Laboratory of Cognition and Personality (SWU), Ministry of Education Chongqing, China; ^2^Faculty of Psychology, Southwest University Chongqing, China; ^3^School of Foreign Languages, Southwest University of Political Science and Law Chongqing, China; ^4^School of Management, Zunyi Medical University Guizhou, China

**Keywords:** bistable perception, Rubin face–vase illusion, task-related fMRI, multivariate pattern analysis, fusiform face area

## Abstract

When viewing the Rubin face–vase illusion, our conscious perception spontaneously alternates between the face and the vase; this illusion has been widely used to explore bistable perception. Previous functional magnetic resonance imaging (fMRI) studies have studied the neural mechanisms underlying bistable perception through univariate and multivariate pattern analyses; however, no studies have investigated the issue of category selectivity. Here, we used fMRI to investigate the neural mechanisms underlying the Rubin face–vase illusion by introducing univariate amplitude and multivariate pattern analyses. The results from the amplitude analysis suggested that the activity in the fusiform face area was likely related to the subjective face perception. Furthermore, the pattern analysis results showed that the early visual cortex (EVC) and the face-selective cortex could discriminate the activity patterns of the face and vase perceptions. However, further analysis of the activity patterns showed that only the face-selective cortex contains the face information. These findings indicated that although the EVC and face-selective cortex activities could discriminate the visual information, only the activity and activity pattern in the face-selective areas contained the category information of face perception in the Rubin face–vase illusion.

## Introduction

The human brain addresses the three-dimensional world by constructing visual representations of two-dimensional retinal information (Chen et al., 2012). However, there are many occasions in life during which conscious percepts might alternate between two or more different interpretations of the visual inputs (Sterzer et al., 2009), such as structure-to-motion objects (Kanai et al., 2010; Knapen et al., 2011; Megumi et al., 2015), ambiguous figures (Sterzer et al., 2002; Wang et al., 2013), and perceptual rivalry (Kanai et al., 2011). For example, conscious percepts might spontaneously switch between a face and a vase when a subject views an ambiguous image, such as the Rubin face–vase illusion (De Graaf et al., 2011). Because most of these images lead to two exclusive perceptual states, they are termed bistable perceptions (Necker, 1832; Rubin, 1915; Eagleman, 2001).

In bistable perception, perceptual states alter between one object and another object originating from complex brain activities (Logothetis et al., 1996; Lumer et al., 1998; Leopold and Logothetis, 1999; Blake and Logothetis, 2002; Long and Toppino, 2004; Lee et al., 2007; Britz et al., 2009; Dijkstra et al., 2016). The Rubin face–vase illusion offers a potential method of investigating the complex neural mechanisms underlying bistable perception (Andrews et al., 2002; Haynes and Rees, 2005; Qiu et al., 2009; Wang et al., 2013).

The activation of the visual cortex was found closely related to perception of the Rubin illusion (Andrews et al., 2002; Hesselmann et al., 2008). Specifically, in a task-related functional magnetic resonance imaging (fMRI) study, the authors showed that the face-selective regions in the fusiform gyrus responded more strongly during the perception of a face (Andrews et al., 2002). Indeed, the perceptual alternations that the subjects experienced when viewing ambiguous figures could activate higher areas of the visual cortex that shared some common features with the perception of binocular rivalry (Tong et al., 1998; Polonsky et al., 2000; Tong and Engel, 2001).

Activities in high-level areas such as the right superior parietal lobule (rSPL) preceded the perceptual alteration of the Rubin face–vase illusion (Britz et al., 2009). Activity in these high-level areas, such as the frontal and parietal brain regions, correlated with switched percepts during bistable perception, as demonstrated by neuroimaging (Kleinschmidt et al., 1998; Sterzer and Kleinschmidt, 2007; Knapen et al., 2011), transcranial magnetic stimulation (TMS) (Carmel et al., 2010; Zaretskaya et al., 2010), and lesion studies (Ricci and Blundo, 1990; Meenan and Miller, 1994; Windmann et al., 2006). Patients with frontal cortex lesions generally exhibited more difficulty shifting from one perception of an ambiguous figure to another compared with patients with more posterior lesions and control subjects (Ricci and Blundo, 1990).

Modulation of the visual cortex from high-level areas also influenced perceptual alternations during the Rubin illusion perception (Kleinschmidt et al., 1998; Qiu et al., 2009; Wang et al., 2013). These results are consistent with other bistable perception studies (Sterzer et al., 2002; Sterzer et al., 2009; Weilnhammer et al., 2013). For example, in binocular rivalry, the frontal and extrastriate regions were related to alternations in perceptual stability (Lumer et al., 1998). In addition, there is increasing evidence supporting the involvement of both the sensory cortex and high-level brain areas in the processing of bistable perception (Blake, 1989; Hsieh et al., 2006; Doesburg et al., 2009; Kanai et al., 2011; Baker et al., 2015; Brascamp et al., 2015; Russo and De Pascalis, 2016).

Studies of the Rubin illusion have greatly advanced our understanding of the complex neural mechanisms underlying bistable perception through univariate (Kleinschmidt et al., 1998; Andrews et al., 2002; Sterzer and Kleinschmidt, 2007) and multivariate analyses (Haxby et al., 2001; Haynes and Rees, 2005; Wang et al., 2013). However, no studies have investigated the issue of category selectivity, that is, whether the perceptual process of illusory face perception is the same to that of illusory vase perception. Although Andrews et al. (2002) found that awareness only changed the responses in the fusiform area, this study did not explore the issue of category selectivity in different visual cortices during perception of the Rubin face–vase illusion, and therefore, we questioned whether different visual cortex areas selectively respond to face or vase percepts.

In the current study, we investigated human cortical activation related to the Rubin face–vase illusion. Using multivariate pattern analysis, more information presented in the multivariate pattern of responses to visual stimuli could be studied compared to univariate analysis; therefore, we could observe more activated areas than possible using univariate analysis. Thus, we used both amplitude and multivariate pattern analyses in this study. We hypothesized that regions of interest (ROIs) (face-selective regions) could discriminate between the different perceptual states of bistable perception and would contain category information about the content of the Rubin face–vase illusion.

## Materials and Methods

### Subjects

Twenty-two (18 women, 18–25 years of age) healthy graduate students from Southwest University were paid to participate in this experiment. All subjects were right-handed and had normal or corrected-to-normal vision. The participants were screened to confirm healthy development using a self-report questionnaire before undergoing scanning. Participants with a history of psychiatric or neurological disorders, those who had received mental health treatment or those who had taken psychiatric medications were excluded. All participants were graduates or undergraduates at Southwest University. Informed written consent was obtained from each participant. The Brain Imaging Center Institutional Review Board of Southwest China University approved this study. The consent and experiment procedures were performed in accordance with the World Medical Association Code of Ethics (the Declaration of Helsinki).

### Experimental Task

In this study, all stimuli were back-projected through a DLP video projector (Tokyo, Japan) (refresh rate: 60 Hz; spatial resolution: 1024 × 768) onto a translucent screen placed inside the fMRI scanner bore. The subjects viewed the stimuli through a mirror located above their eyes. The viewing distance was 83 cm. The tasks were completed on 2 separate days: on day 1, the retinotopic visual areas and the localizer (ROIs) were scanned, and on day 2, the ambiguous (Rubin face–vase illusion task) and unambiguous conditions were scanned.

#### The Retinotopic Visual Area Scanning

To define the visual cortex boundaries, retinotopic mapping scanning was used, according to a standard phase-encoded method developed by Sereno et al. (1995) and Engel et al. (1997). The subjects viewed a rotating wedge and expanding ring stimuli that created traveling waves of neural activity in the visual cortex. The scan was only used to define the boundary of the early visual cortex, and the retinotopic mapping data were not further used.

#### The Localizer Scanning

The fMRI tests also contained a block-design run to localize the ROIs, including the face-selective areas. The subjects viewed images of faces, non-face objects, and texture patterns (scrambled faces), which subtended 6.2 × 6.2° and were presented at the center of the screen. The images appeared at a rate of 2 Hz in blocks of 12 s, interleaved with 12-s blank blocks. Each image was presented for 300 ms, followed by a 200-ms blank interval. Each block type was repeated five times in the run, which lasted 360 s. The subjects performed a one-back task during which they pressed a key to indicate the same images. This scanning enabled us to define all ROIs in the visual cortex from V1 to higher level areas.

#### Ambiguous Condition

**FIGURE 1:**
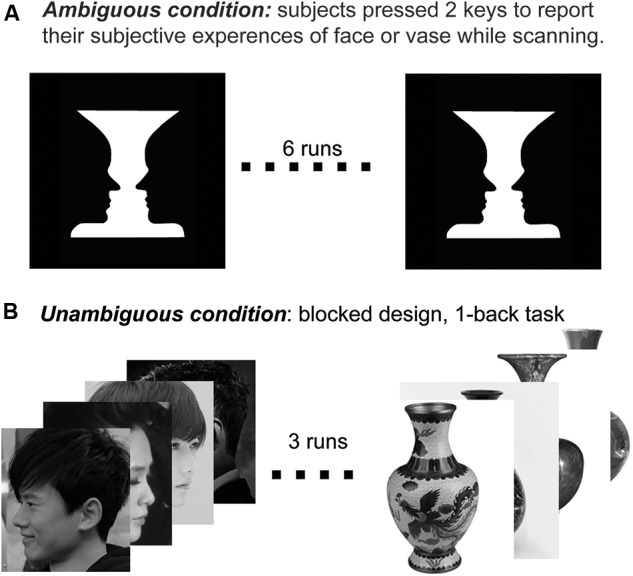
**(A)** An illustration of the ambiguous condition. First, a red circle (dot) was represented centrally on the screen for 6 s to prepare. Second, the Rubin face–vase illusion was viewed for 276 s, and the subjects had to keep pressing one of the two buttons to indicate their perceptions. Third, for another 6 s, a red circle dot was placed in the middle of the screen until the end of a run. The ambiguous condition comprised six runs in total, and the subjects were instructed to keep their heads still throughout the scanning of the entire run. **(B)** An illustration of the unambiguous condition. Subjects were presented with alternating blocks of faces or vases, with 24 images per block interleaved with 12-s blank blocks. Every photograph was presented for 300 ms, followed by a 200 ms blank interval. Each block type was repeated six times in the run, and the entire design required 300 s of scanning in total. The sequences of the representations of face blocks and vase blocks were random. The participants were instructed to pay attention to the fixation, and they performed a one-back task.

In the scanner, the participants were presented the Rubin face–vase illusion. They were instructed to fixate on a small red circle dot in the middle of the Rubin face–vase stimulus and to indicate any perceptual alternations between the face and vase by continuously pressing one of the two buttons as soon as the new perception was perceived. There were three phases in each “Rubin face–vase illusion” run. First, a red circle dot was represented centrally on the screen for 6 s to prepare. Second, the Rubin face–vase illusion was viewed for 276 s, and the subjects had to keep pressing one of the two buttons to indicate their perceptions. Third, for another 6 s, a red circle dot was placed in the middle of the screen until the end of a run. The ambiguous condition comprised six runs in total, and the subjects were instructed to keep their heads still throughout the entire scanning of the runs (**Figure 1A**).

#### Unambiguous Condition

In the unambiguous condition, the stimuli were grayscale photos of faces and vases (6.2 × 6.2°). Examples are shown in **Figure 1B**. The photographs were unfamiliar to all participants. During the scanning of the unambiguous condition, a white dot was represented centrally on the black screen, and the images appeared at a rate of 2 Hz in blocks of 12 s, interleaved with 12-s blank blocks. The subjects were represented with alternating blocks of faces or vases with 24 images per block, interleaved with 12-s blank blocks. Every photograph was presented for 300 ms, followed by a 200-ms blank interval. Each block type was repeated six times in the run, and the entire design required a total of 300 s scanning. The sequences of the face block and vase block representations were random. The participants were instructed to attend the fixation and to perform a one-back task. Each subject participated in three unambiguous condition runs.

### MRI Data Acquisition

For each participant, high-resolution T1-weighted structural images were acquired. A 3-T Siemens Trio MRI scanner (Siemens Medical, Erlangen, Germany) was used to collect all images. fMRI data were collected using the scanner with a 12-channel phase-array coil. Blood oxygenation level dependent (BOLD) signals were measured with an EPI sequence (TR: 2000 ms; TE: 30 ms; FOV: 192 mm × 192 mm; matrix: 64 × 64; flip angle: 90; slice thickness: 3.0 mm; gap: 0 mm; number of slices: 33; slice orientation: axial). The bottom slice was positioned at the bottom of the temporal lobes. A three-dimensional MPRAGE structural data set (resolution: 1 mm × 1 mm × 1 mm; TR: 2000 ms; TE: 3.02 ms; FOV: 256 mm × 224 mm; flip angle: 8; number of slices: 176; slice orientation: sagittal) was collected before the task-related functional runs.

### Functional MRI Data Analysis

#### The Preprocessing Process

The anatomical volume for each subject was first aligned according to the AC-PC locations and then transformed into Talairach coordinates. Functional volumes in all ambiguous, unambiguous, localizer, and retinotopic visual areas scanning were preprocessed using BrainVoyager QX, including three-dimensional motion correction, linear trend removal, and high-pass (0.015 Hz) filtering. Head motion within any fMRI test was less than 3 mm for all subjects. The functional volumes were then aligned to the anatomical volume. The first 6 s of BOLD signals was discarded to minimize any transient magnetic saturation effects.

#### Defining Region of Interests (ROIs)

A general linear model (GLM) procedure was applied to the retinotopic visual area scanning and localizer runs, and seven ROIs were defined from the early to late visual cortices. Face-selective regions were defined as regions that responded more strongly to faces than to non-face objects (*p* < 0.001, uncorrected). These regions included the averaged left occipital face area (lOFA) (-37, -72, -14; *SD* = 7, 8, 5) in Talairach coordinates, as well as the rOFA (33, -68, -13; *SD* = 4, 8, 4), the left fusiform face area (lFFA) (-43, -50, -16; SD = 3, 6, 4), the rFFA (37, -50, -15; *SD* = 5, 7, 3), the left superior temporal sulcus (lSTS) (-50, -61, 12; *SD* = 4, 12, 4), and the rSTS (45, -53, 9; *SD* = 4, 11, 4). The responsive area (V1) in the early visual cortex (EVC) was defined as the area that responded more strongly to scrambled faces than to a blank screen (*p* < 0.001, uncorrected), V1 (-2, -91, -6; *SD* = 4, 2, 4). The EVC boundaries were delineated with a standard retinotopic method. All coordinates represented in the paper are the average values from all subjects.

#### Activity Amplitude Analysis

For the unambiguous condition, data were first extracted from each of the ROIs. Then, the activity amplitude in each ROI was used as the estimated beta value through a GLM procedure.

For the ambiguous condition, the event-related averaging method was used. Event-related BOLD signals were calculated separately for each ROI for each subject and condition, following the method used by Larsson et al. (2006), Liu et al. (2007), and Fang et al. (2009). For each fMRI run, the time course of MR signal intensity was first extracted by averaging the data across all voxels within the pre-defined ROI and then normalized with the mean intensity across the run. The maximum BOLD signal during the test stimulus was taken as the measure of response amplitude for each condition in subsequent analyses.

#### Activity Pattern Analysis

The multivariate pattern analysis introduced in the analysis of the ambiguous and unambiguous conditions was a standard correlation analysis of spatial activity pattern, as used by Haxby et al. (2001). For each ROI and each run, beta values were estimated from the time course of the BOLD signal in each voxel. Spatial response patterns (i.e., spatial activity patterns) were then extracted for the face and vase. Within each ROI, we computed the correlation coefficient between the spatial activity patterns evoked by the face or vase in different runs, and then we transformed these coefficients to Fisher *z* scores. These transformed coefficients were averaged across possible run combinations. In the ambiguous condition, we defined the discrimination index = (Corr[face]+Cor[vase])/2 – Corr (face, vase). The result indicated that the activity pattern in the face and vase perception was significantly different if this index was significantly above zero. Similar to previous studies (Peelen et al., 2009), we also calculated the face category information = Corr (Amb face, Unamb face) – Corr (Amb face, Unamb vase). A significantly positive index indicated that the activity pattern induced by bistable face perception was more similar to the unambiguous face than to the unambiguous vase.

## Results

### Behavioral Results

**FIGURE 2:**
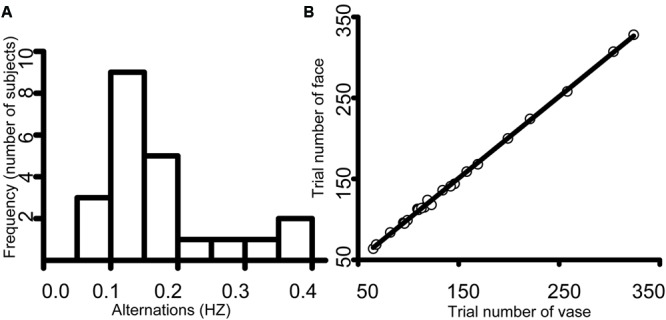
**(A)** An illustration of the frequency histogram. The average face alternation was approximately 0.14 Hz, with a standard deviation of 0.11 Hz, and the mean vase alternation was 0.16 Hz (*SD* = 0.12). **(B)** An illustration of the scatter diagram. The trial number for face perception ranged from 64 to 328 (mean = 148.5, *SD* = 72.55), which was approximately the same as the trial number of vase perception (range = 65–324, mean = 147.7, *SD* = 71.83).

There was substantial variability in the mean alternation frequency for each subject. The frequency histogram showed the number of participants in the different reported perceptual alternations (**Figure 2A**). The average face switch frequency was approximately 0.14 Hz, with a standard deviation of 0.11 Hz, and the mean vase switch frequency was 0.16 Hz (*SD* = 0.12). The trial number for face perception ranged from 64 to 328 (mean = 148.5, *SD* = 72.55), which was nearly the same as the vase perception trial number (range = 65–324, mean = 147.7, *SD* = 71.83) (**Figure 2B**), supporting the effectiveness of our study.

### Activity Amplitude Analysis Results

**FIGURE 3:**
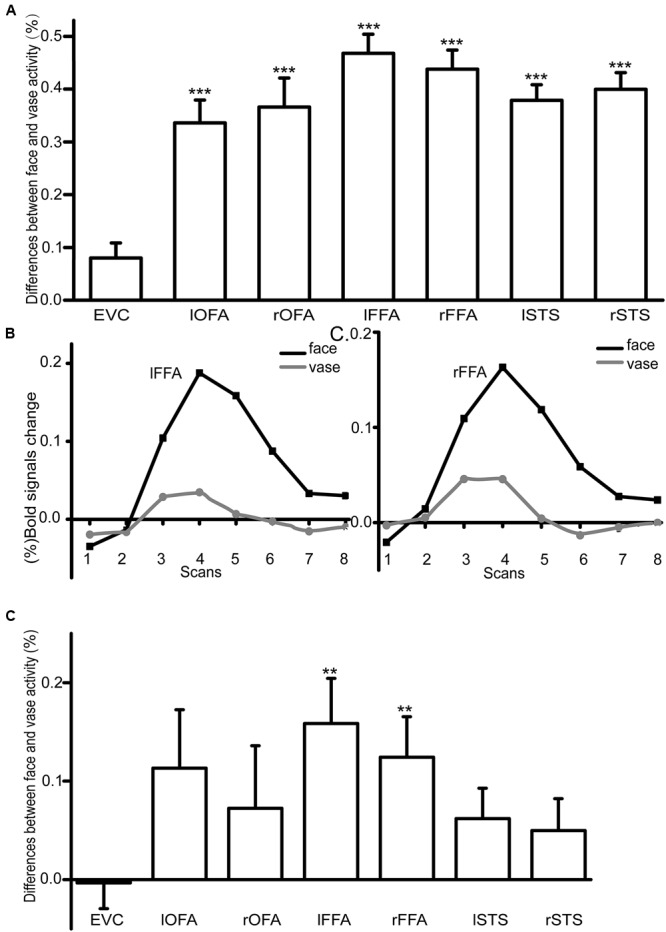
**(A)** An illustration of the difference between face and vase perception activity amplitude in the unambiguous condition (^∗∗^*p* < 0.01, ^∗∗∗^*p* < 0.001, Bonferroni corrected). **(B,C)** The activity amplitude differences in FFA between the face and vase in the ambiguous condition. The *X*-axis represented the eight TR′ scans in lFFA and rFFA separately when viewing the Rubin face–vase illusion in the scanner, and the *Y*-axis represented the (%) BOLD signals change. **(D)** An illustration of the difference between face and vase perception activity amplitude in the ambiguous condition (^∗∗^*p* < 0.01, ^∗∗∗^*p* < 0.001, Bonferroni corrected).

We measured the BOLD signals in response to the face and vase perception in six ambiguous fMRI runs and three unambiguous fMRI runs. As expected, in the unambiguous condition, the differences (face–vase) between the face and vase activity amplitudes were significant in the bilateral OFA (lOFA: *T* = 7.70, *p* = 1.50^∗^10^-7^, Bonferroni corrected; rOFA: *T* = 6.70, *p* = 1.26^∗^10^-6^, Bonferroni corrected), the FFA (lFFA: *T* = 12.98, *p* = 1.69^∗^10^-11^, Bonferroni corrected; rFFA: *T* = 12.05, *p* = 6.75^∗^10^-11^, Bonferroni corrected), and the STS (lSTS: *T* = 12.84, *p* = 2.06^∗^10^-11^, Bonferroni corrected; rSTS: *T* = 12.59, *p* = 3.00^∗^10^-11^, Bonferroni corrected) (**Figure 3A**), but not in the EVC (*T* = 2.81, *p* = 0.01, Bonferroni corrected). For the ambiguous condition, the time courses of the BOLD signals in the bilateral FFA are shown in **Figures 3B,C**. We extracted the amplitude for each condition in each ROI. The results showed that only the bilateral FFA had significantly higher responses during the face perception than the vase perception after Bonferroni multiple comparisons correction (lFFA: *T* = 3.46, *p* = 0.002, Bonferroni corrected; rFFA: *T* = 3.02, *p* = 0.006, Bonferroni corrected) (**Figure 3D**).

### The Correlation between Neural Activity and Perceptual Duration

We then investigated the correlation between neural activities and face perceptual duration in all seven ROIs (ambiguous condition). We found the activity amplitudes to face perception of the lOFA (*r* = 0.63, *p* = 0.01, Bonferroni corrected), the bilateral FFA (lFFA: *r* = 0.69, *p* = 0.002, Bonferroni corrected; rFFA: *r* = 0.75, *p* = 0.0003, Bonferroni corrected), and the bilateral STS (lSTS: *r* = 0.63, *p* = 0.01, Bonferroni corrected; rSTS: *r* = 0.69, *p* = 0.003, Bonferroni corrected) were significantly positively correlated with the face perceptual duration.

According to the results, the higher activity amplitude of the face-selective areas might imply that a longer face perception duration leads to lower alternation frequency. However, this correlation might be inconclusive. The correlation might be an artifact of the experimental design because the time of perceptual duration *per se* could lead to increased BOLD responses.

### Activity Pattern Analysis Results

**FIGURE 4:**
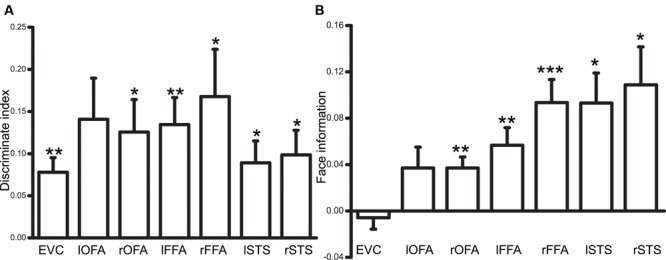
**(A)** An illustration of the discrimination index result (^∗^*p* < 0.05, ^∗∗^*p* < 0.01, ^∗∗∗^*p* < 0.001, Bonferroni corrected). **(B)** An illustration of the results of the face information index for category information (^∗^*p* < 0.05, ^∗∗^*p* < 0.01, ^∗∗∗^*p* < 0.001, Bonferroni corrected).

We first calculated the correlations between the spatial patterns of activities corresponding to ambiguous face and vase perception. Then, the discrimination index was defined as the difference between the correlation coefficients calculated from the same category (face vs. face or vase vs. vase) and from different categories (face vs. vase). A significantly positive index demonstrated that a specific brain region could discriminate the different perceptual states of a subject. As shown in **Figure 4A**, the EVC and face-selective ROIs showed a significant discrimination index (bilateral FFA, STS, and right OFA: *p* < 0.05, Bonferroni corrected; lOFA: *p* = 0.06, Bonferroni corrected), which suggested that the neural activity across the specialized visual cortex could be affected by visual awareness changes. However, it is still unknown whether each area can discriminate between conscious face perception and vase perception. We then calculated the category information in each ROI. This information represents the similarity of the activity pattern corresponding to illusory face perception and real face perception relative to real vase perception. **Figure 4B** illustrates that the face information from the face-selective visual areas was only significant after Bonferroni multiple comparisons correction was performed (*p* < 0.05) (lOFA: *p* = 0.3).

## Discussion

In the current study, we systematically investigated the neural mechanisms underlying the Rubin face–vase illusion. During unambiguous face perception, the face-selective areas, including the bilateral OFA, the FFA, and the STS, were significantly activated. However, the results of the ambiguous condition indicated that only the bilateral FFA responded significantly to face perception when viewing the Rubin face–vase illusion.

The amplitude analysis results revealed that only the activities of the bilateral FFA were significantly higher during face perception than during vase perception in the ambiguous condition, which was consistent with previous findings (Hasson et al., 2001; Andrews et al., 2002; Wang et al., 2013). It is widely known that the OFA, the FFA, and the STS have been recognized as face-selective regions (Kanwisher et al., 1997; Pitcher et al., 2007; Wang et al., 2013) that respond stronger to faces than objects, as shown in the unambiguous condition of the current study. A previous fMRI study has also reported that the FFA showed similar responses to ambiguous face and vase perceptions (Andrews et al., 2002).

The OFA and STS results might be explained because they selectively responded to some specific attributes of the face that could be absent in the bistable stimulus. For example, the STS was believed to encode the changeable aspects of the face, such as facial expressions and gaze (Haxby et al., 2001). The relationship between the activity amplitude of face-selective areas (the left OFA, bilateral FFA, and bilateral STS) and the duration of face perception revealed the roles of face-selective areas in the maintenance of conscious face perception. This finding was similar to previous findings that the frontal–parietal areas had a close relationship with the perceptual switches (Windmann et al., 2006; Sterzer et al., 2008; Zaretskaya et al., 2010; De Graaf et al., 2011; Knapen et al., 2011; Weilnhammer et al., 2013; Megumi et al., 2015).

Further analysis of the activity patterns showed that not only the face-selective areas (the bilateral FFA, bilateral STS, and right OFA) but also the EVC could discriminate face perception from vase perception in the ambiguous condition. However, only the face perception activity patterns of the face-selective regions (i.e., the right OFA, bilateral FFA, and bilateral STS) showed a greater similarity to the activity patterns induced by the real face than those induced by the real vase. Although the EVC could discriminate different perceptual states of bistable perception, it did not contain any information about the content of the Rubin face–vase illusion.

The results from the discrimination index showed that we could discriminate the two perceptual states based on the activity patterns from both the EVC and the face-selective cortex. This pattern correlation method is similar to the classification method used by Wang et al. (2013). Our results were also consistent with the findings by Wang et al. (2013) indicating that the representation in the EVC changed with changing awareness. The results from the category information analysis supported the finding that the changes of representation in the EVC were not related to the face awareness changes. Thus, the activity pattern changing in the EVC may result from two different mechanisms. First, some relatively low-level properties of the stimulus might be represented in the EVC change with the perceptual state change. For example, Fang et al. (2009) found that border ownership was represented in the human EVC. In our study, it was evident that the border belonged to the face or the vase when the subjects perceived the face or the vase, respectively, which might have resulted in different activity patterns in the EVC. Second, it was found that an object identity may affect the representations of that object in the human EVC (Hsieh et al., 2010), indicating the impact of top-down modulation on the representation of the EVC. The Wang et al. (2013) study also revealed the enhanced top-down influence on bistable perception, further supporting this possibility.

## Conclusion

As we expected, the face-selective regions not only showed discrimination of face and vase perception but also contained face information when the subjects perceived the face. Together, the results of the activity amplitude analysis and activity pattern analysis indicate that both the EVC and high-level face-selective cortex were regulated by conscious awareness. However, only the face-selective cortex was related to the content of the face in the subjective experience. These results strongly support the role of face-selective areas in the conscious processing of faces.

## Author Contributions

TB, NS, and LH responsible for the experiment design, data collecting, and data analyzing and JQ, YZ, and XW responsible for the manuscript preparation.

## Conflict of Interest Statement

The authors declare that the research was conducted in the absence of any commercial or financial relationships that could be construed as a potential conflict of interest.
